# Macrophage migration inhibitory factor induces vascular leakage via autophagy

**DOI:** 10.1242/bio.201410322

**Published:** 2015-01-23

**Authors:** Hong-Ru Chen, Yung-Chun Chuang, Chiao-Hsuan Chao, Trai-Ming Yeh

**Affiliations:** 1 The Institute of Basic Medical Sciences, Medical College, National Cheng Kung University, Tainan, Taiwan; 2 Department of Medical Laboratory Science and Biotechnology, Medical College, National Cheng Kung University, Tainan, Taiwan

**Keywords:** Cytokine, Shock, Autophagy, MIF, Endothelial cells

## Abstract

Vascular leakage is an important feature of acute inflammatory shock, which currently has no effective treatment. Macrophage migration inhibitory factor (MIF) is a pro-inflammatory cytokine that can induce vascular leakage and plays an important role in the pathogenesis of shock. However, the mechanism of MIF-induced vascular leakage is still unclear. In this study, using recombinant MIF (rMIF), we demonstrated that MIF induced disorganization and degradation of junction proteins and increased the permeability of human endothelial cells *in vitro*. Western blotting analysis showed that rMIF treatment induced LC3 conversion and p62 degradation. Inhibition of autophagy with a PI3K inhibitor (3-MA), a ROS scavenger (NAC) or autophagosomal-lysosomal fusion inhibitors (bafilomycin A1 and chloroquine) rescued rMIF-induced vascular leakage, suggesting that autophagy mediates MIF-induced vascular leakage. The potential involvement of other signaling pathways was also studied using different inhibitors, and the results suggested that MIF-induced vascular leakage may occur through the ERK pathway. In conclusion, we showed that MIF triggered autophagic degradation of endothelial cells, resulting in vascular leakage. Inhibition of MIF-induced autophagy may provide therapeutic targets against vascular leakage in inflammatory shock.

## INTRODUCTION

The endothelial barrier is a well-regulated structure that maintains a low and selective permeability to fluid and molecules under normal physiological conditions. Disruption of the cytoskeleton, cell-cell junctions, and cell-to-matrix attachments can cause dysfunction of endothelial barrier, which occurs during exposure to inflammatory cytokines, pathogen infection, or cancer metastasis (Evans and Richardson, 1968; Förster, 2008; Marker et al., 1984; Schnittler et al., 1989; Shen et al., 2010; Spindler et al., 2010). Dysfunction of the endothelial barrier results in vascular leakage, a condition in which the extravasation of fluid, small molecules, pathogens and leukocytes occurs. Vascular leakage can lead to life-threatening dehydration, hypotension, and shock. Due to the complexity of the pathogenesis of vascular leakage, the detailed mechanisms that regulate vascular permeability during shock are still under investigation.

A “cytokine storm” refers to the secretion of large amounts of cytokines into circulation during pathogen infection or sepsis (Pang et al., 2007; Riedemann et al., 2003; Yokota et al., 2000). The uncontrolled release of cytokines leads to dysfunction of the endothelial barrier and an increase in vascular permeability, resulting in vascular leakage. Among these cytokines, macrophage migration inhibitory factor (MIF) has been highlighted as a key player in septic shock and infection (Bernhagen et al., 1994; Delaloye et al., 2012; de Dios Rosado and Rodriguez-Sosa, 2011). Blockage of MIF increased the survival rate in a mouse model of sepsis (Al-Abed et al., 2005; Bozza et al., 1999; Chagnon et al., 2005). Recently, an elevated level of MIF was also observed during infections with viruses, such as the dengue virus and Ebola virus (Assunção-Miranda et al., 2010; Wauquier et al., 2010). In addition to its role in infection, MIF also plays a role in cardiovascular diseases, as elevated levels of MIF were observed in patients with these diseases (Müller et al., 2012; Zernecke et al., 2008). MIF is also known as glycosylation-inhibiting factor, L-dopachrome isomerase, or phenylpyruvate tautomerase, and it can be secreted by a wide variety of cells, including macrophages, hepatocytes, and endothelial cells, upon stimulation. Once MIF binds to its receptors CXCR2, CXCR4 and/or CD74 (Bernhagen et al., 2007; Schwartz et al., 2009), downstream signal phosphoinositide 3-kinase (PI3K)/Akt or mitogen-activated protein kinase (MAPK)/extracellular signal-regulated kinase (ERK) is activated, mediating the inflammatory response (Lue et al., 2006; Lue et al., 2007). Our previous study revealed that MIF is involved in vascular leakage during dengue virus infection (Chuang et al., 2011). However, it is not yet fully understood how MIF disrupts the endothelial barrier.

Recently, we found that MIF can induce autophagy in hepatocytes via the generation of reactive oxygen species (ROS) (Chuang et al., 2012). Autophagy is a self-destructive mechanism that regulates the turnover of intracellular organelles and macromolecules. This pathway begins with the formation of double-membrane-bound autophagosomes that are induced by starvation, ROS, endoplasmic reticulum stress and viral infection. During the late stage, the double-membrane autophagosomes fuse with lysosomes to form autophagolysosomes, which mediates the degradation of their contents. This process is involved in the pathogenesis of many diseases, including pathogen infection, metabolic and neurodegenerative disorders, cardiovascular and pulmonary diseases, and cancer (Chen et al., 2008; Hara et al., 2006; Li et al., 2013; McLean et al., 2011; Niu et al., 2012; Singh et al., 2009). It was also reported that autophagy is involved in the disruption of the endothelial barrier in the blood-brain-barrier upon stimulation with nanoalumina (Chen et al., 2013). Given that MIF may play a role in the induction of autophagy and autophagy may relate to dysfunction of the endothelial barrier, we rationalized that MIF may induce endothelial dysfunction through autophagy, resulting in vascular leakage.

In this study, we used recombinant MIF (rMIF) to demonstrate that MIF can induce autophagy in endothelial cells and cause an increase in vascular permeability in both *in vitro* cell culture and *in vivo* mice experiments. This process is blocked by an ERK inhibitor. The results of this study provide a potential mechanism for how MIF induces vascular leakage and suggest that autophagy has an important role in regulating endothelial barrier function.

## RESULTS

### MIF disrupts endothelial barrier function through deconstruction of cell-cell junctions

**Fig. 1.**
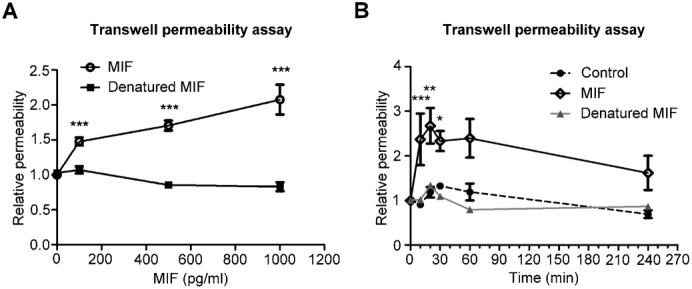
MIF increases the permeability of endothelial cells. (A) HMEC-1 cells were treated with different doses of rMIF for 30 min, and endothelial permeability was determined using a transwell permeability assay. (B) HMEC-1 cells were treated with or without 1 ng/ml rMIF, and endothelial permeability was determined using a transwell permeability assay at the indicated time points. *P<0.05, **P<0.01, ***P<0.001.

The effect of MIF on endothelial barrier function was measured using human microvascular endothelial cell line (HMEC-1) monolayers under basal and rMIF-treated conditions. Low-dose treatment of monolayers with rMIF (100 pg/ml) increased endothelial permeability, and the permeability was positively correlated with the dosage (Fig. 1A). rMIF at a dose of 1 ng/ml increased endothelial permeability in 10 min, and this effect persisted for 4 h (Fig. 1B).

**Fig. 2.**
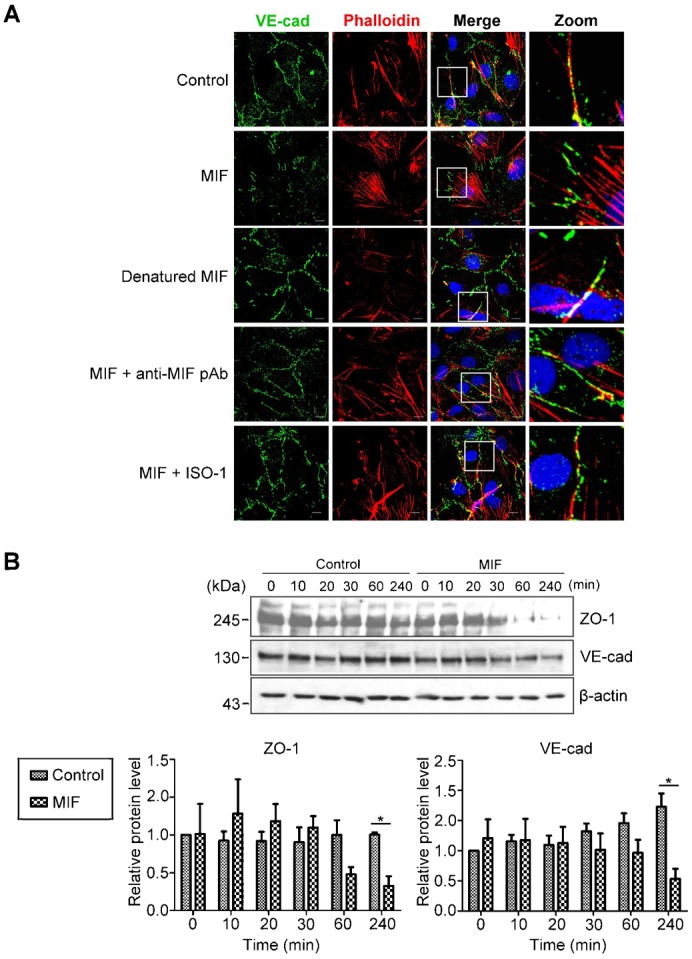
MIF disrupts the arrangement and decreases the level of junction proteins in endothelial cells. (A) HMEC-1 cells were treated with 1 ng/ml rMIF, rMIF with 50 nM ISO-1, rMIF with 1 mg/ml anti-MIF polyclonal antibody or heat-denatured rMIF. An immunofluorescence assay was applied after fixation with 4% paraformaldehyde. Confocal images were acquired with an FV1000 confocal microscope (Olympus). Bars: 10 µm. (B) HMEC-1 cells were treated with or without 1 ng/ml rMIF for the indicated time periods. Relative ZO-1 and VE-cadherin protein levels were determined by western blotting with specific antibodies.

To investigate whether the increase in endothelial permeability was the result of junction protein disorganization, the distribution of the filamentous actin cytoskeleton and the junction protein VE-cadherin in HMEC-1 cells were visualized with immunofluorescence staining. Treatment of HMEC-1 cells with rMIF decreased junction-localized VE-cadherin and altered the actin cytoskeleton from the cortical actin ring to stress fibers (Fig. 2A). Pre-incubation of rMIF with a MIF inhibitor (S,R)-3-(4-hydroxyphenyl)-4,5-dihydro-5-isoxazole acetic acid methyl ester (ISO-1) or anti-MIF polyclonal antibodies attenuated this effect. In addition, heat-denatured rMIF did not induce VE-cadherin translocation or stress fiber formation (Fig. 2A). Instability of cell-cell junctions usually results from the degradation of junction proteins. Therefore, we also measured the relative protein levels of zonula occludens protein-1 (ZO-1) and VE-cadherin in HMEC-1 cells. Western blotting analysis of ZO-1 and VE-cadherin revealed that rMIF decreased the levels of ZO-1 and VE-cadherin in HMEC-1 cells within 4 h (Fig. 2B). Taken together, these data demonstrated that MIF disrupted endothelial barrier function by promoting the translocation and degradation of junction proteins.

### MIF induces autophagy formation by inhibiting mTOR signaling in endothelial cells

**Fig. 3.**
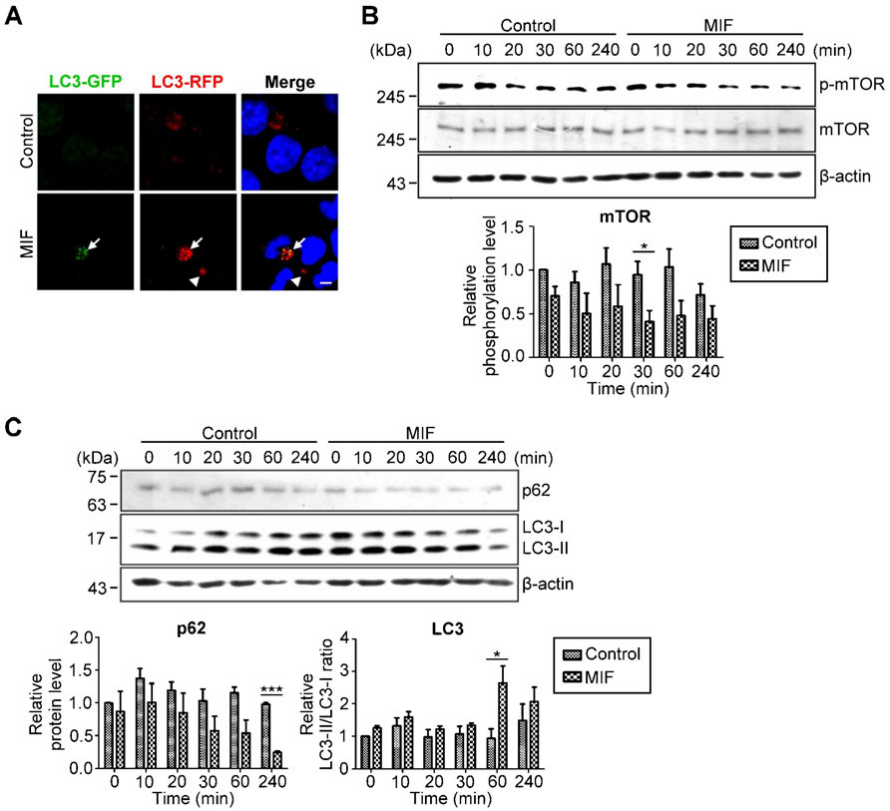
MIF facilitates autophagy induction in endothelial cells. (A) HMEC-1 cells were transfected with ptfLC3 and subsequently treated with or without 1 ng/ml rMIF for 1 h. Confocal images were obtained using an FV1000 confocal microscope (Olympus). Arrows, autophagosomes. Arrowheads, autophagolysosomes. Bar: 10 µm. (B) HMEC-1 cells were treated with or without 1 ng/ml rMIF for the indicated time periods. Relative levels of p-mTOR/mTOR and (C) p62, LC3-I and LC3-II were determined by western blotting with specific antibodies.

A previous study revealed that MIF induces autophagy through ROS generation in hepatoma cells (Chuang et al., 2012). It has also been indicated that autophagy promotes hepatocellular carcinoma cell invasion through down-regulation of E-cadherin (Li et al., 2013). Accordingly, we proposed that autophagy also mediates the down-regulation of ZO-1 and VE-cadherin in MIF-activated endothelial cells. Therefore, our first step was to confirm whether MIF promotes autophagy induction in endothelial cells. To visualize autophagy induction, the mRFP-GFP tandem fluorescence-tagged LC3 plasmid (ptfLC3) (Kimura et al., 2007) was applied. Because EGFP and mRFP show differential sensitivity to low pH levels, the EGFP signal is diminished in the acidic environment of lysosomes, while the mRFP signal is not affected. Confocal images showed that rMIF induced the formation of punctate areas of red fluorescence, indicating that MIF induced autophagolysosome formation after 1 h of incubation with endothelial cells (Fig. 3A). Target of rapamycin (TOR) has long been known to be a key regulator of autophagy (Noda and Ohsumi, 1998). Western blotting showed that rMIF treatment down-regulated the phosphorylation level of mTOR (Fig. 3B), indicating that the signaling pathway that suppresses the induction of autophagy was inhibited. Degradation of p62 and conversion of LC3-I to LC3-II also revealed that rMIF induced autophagy within 30 min, and the decrease in LC3-I and LC3-II after 240 min of rMIF treatment indicated the occurrence of autophagic degradation (Fig. 3C).

### Autophagy mediated MIF-induced dysfunction of the endothelial barrier

**Fig. 4.**
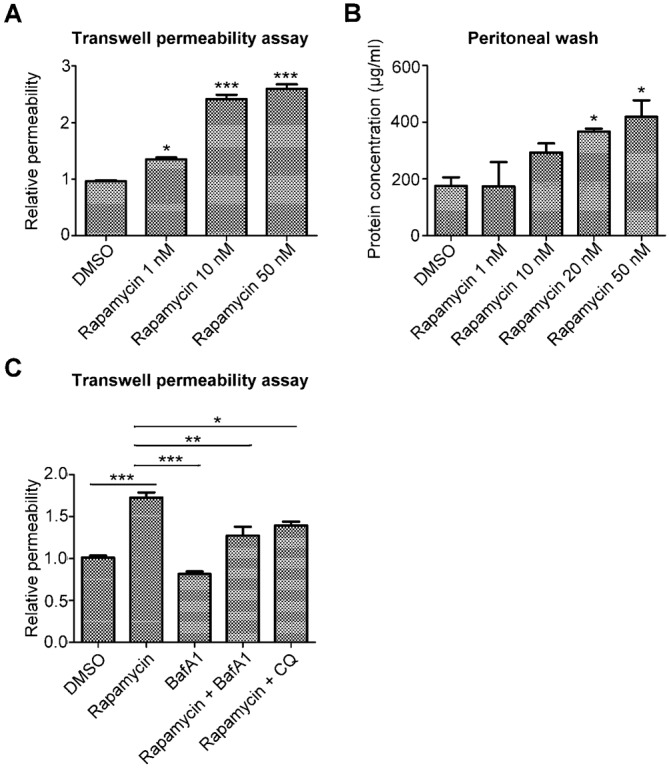
Rapamycin induces autophagy and vascular leakage. (A) HMEC-1 cells were seeded on transwell inserts and subsequently treated with the indicated concentrations of rapamycin for 30 min. The endothelial permeability was determined using a transwell permeability assay. (B) BALB/c mice were intraperitoneally injected with DMSO and the indicated concentrations of rapamycin. After 30 min, the mice were sacrificed, and the abdominal cavity was washed with 10 ml of PBS. The concentration of protein in the abdominal washings was determined using the BCA method. (C) HMEC-1 cells were treated with rapamycin with or without BafA1 or CQ for 30 min, and the endothelial permeability was determined using a transwell permeability assay. *P<0.05, **P<0.01, ***P<0.001.

Although some studies have suggested that autophagy might disrupt cell-cell junctions (Chen et al., 2013; Li et al., 2013), limited studies have shown that autophagy can directly induce endothelial barrier dysfunction. As a result, we used the mTOR inhibitor rapamycin to induce autophagy in HMEC-1 cells. Our results showed that rapamycin induced endothelial barrier dysfunction in a dose-dependent manner (Fig. 4A). This effect was confirmed by *in vivo* experiments examining protein extravasation in the abdominal cavity (Fig. 4B). We further hypothesized that autophagic degradation may also be involved in autophagy-induced vascular leakage. Thus, we attempted to rescue endothelial barrier dysfunction by inhibiting the fusion of autophagosomes and lysosomes. Blocking autophagolysosome formation using bafilomycin A1 (BafA1) and chloroquine (CQ) fully or partially abrogated MIF-induced endothelial hyperpermeability, indicating that rapamycin-induced vascular leakage is at least partially reliant on autophagic degradation (Fig. 4C).

**Fig. 5.**
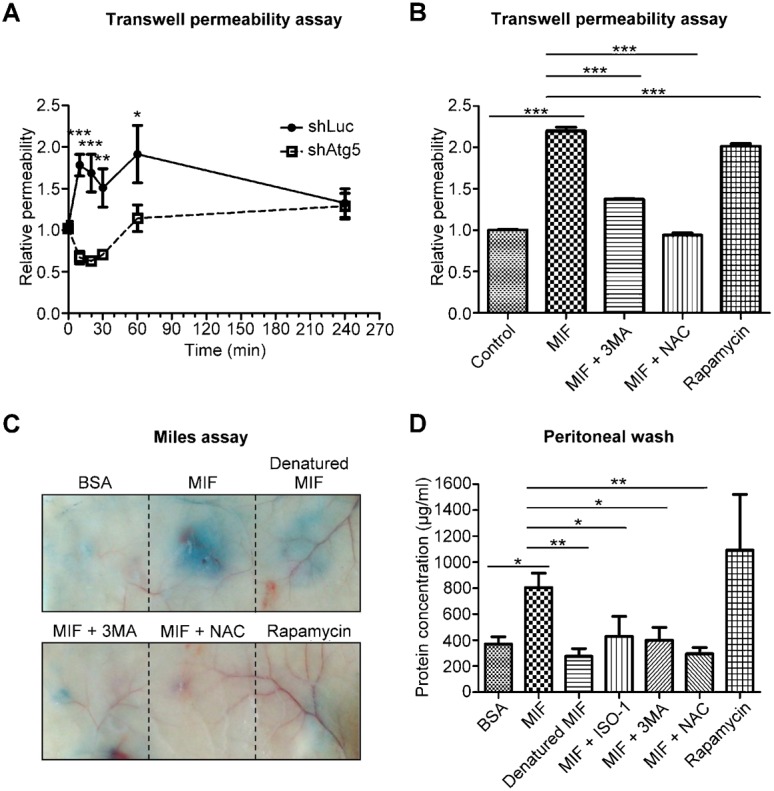
MIF increases vascular permeability through autophagy. (A) HMEC-1 cells were transfected with luciferase and Atg5 shRNA. After selection with puromycin, the resulting stable clones were treated with 1 ng/ml rMIF for the indicated time periods, and the endothelial permeability was determined using a transwell permeability assay with streptavidin-HRP and TMB. (B) HMEC-1 cells were treated with 1 ng/ml rMIF, MIF and 3-MA, rMIF and NAC, or rapamycin for 30 min. Endothelial permeability was determined using a transwell permeability assay. (C) BALB/c mice were subcutaneously injected with BSA, rMIF, heat-denatured rMIF, rMIF and 3-MA, rMIF and NAC, or rapamycin. Evans blue dye (5%, 200 µl) was intravenously injected into the mice, which were sacrificed 30 min after dye injection. (D) BALB/c mice were intraperitoneally injected with 0.1 mg/ml BSA, 0.1 mg/ml murine rMIF, rMIF and ISO-1, rMIF and 3-MA, rMIF and NAC, or rapamycin. After 30 min, the mice were sacrificed, and the abdominal cavities were washed with 10 ml of PBS. The concentration of protein in abdominal washings was determined using the BCA method. n = 4, *P<0.05, **P<0.01, ***P<0.001.

To confirm the role of autophagy in MIF-induced vascular leakage, we utilized shRNA and small molecule inhibitors to inhibit autophagy induction. A stable clone of Atg5-depleted HMEC-1 cells (shAtg5) was acquired and compared with control HMEC-1 cells (shLuc). *In vitro* experiments revealed that rMIF increased the permeability of shLuc cells within 10 min, while the barrier function of shAtg5 cells was not significantly altered (Fig. 5A). The PI3K inhibitor 3-methyladenine (3-MA) and the ROS scavenger N-acetyl-L-cysteine (NAC) are common molecules used to prevent autophagy. Co-treatments including rMIF together with 3-MA or NAC effectively attenuated the rMIF-induced increase in permeability (Fig. 5B). On the contrary, inducing autophagy with rapamycin alone caused an increase in permeability (Fig. 5B).

To test whether 3-MA and NAC also reversed MIF-induced vascular leakage *in vivo*, the Miles assay and the peritoneal wash method were applied. Subcutaneous injection of rMIF increased the leakage of Evans blue dye from capillaries, while bovine serum albumin (BSA) and heat-denatured rMIF did not have similar effects (Fig. 5C). Co-injection of 3-MA or NAC together with rMIF abolished MIF-induced vascular leakage (Fig. 5C), indicating that this effect was mediated by autophagy. Intraperitoneal injection of rMIF increased the protein concentration in the peritoneal cavity (Fig. 5D), indicating an increase in vascular permeability and protein extravasation. The effect of rMIF on peritoneal protein extravasation was inhibited in the presence of ISO-1 or when denatured rMIF was used (Fig. 5D).

### Autophagic degradation is responsible for MIF-induced vascular leakage

**Fig. 6.**
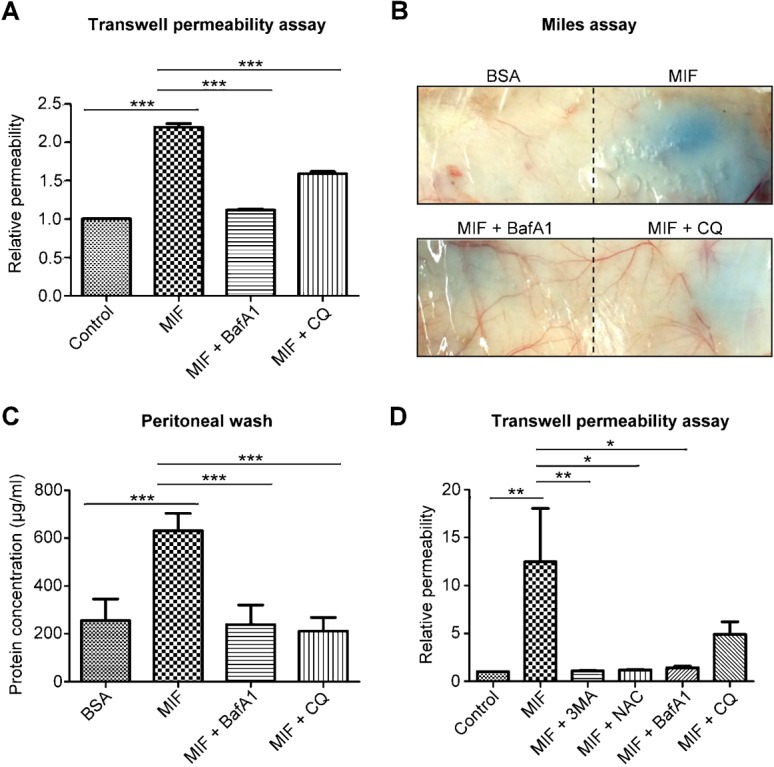
Inhibition of autophagic degradation rescues MIF-induced vascular leakage. (A) HMEC-1 cells were treated with rMIF, rMIF and BafA1, or rMIF and CQ for 30 min. The endothelial permeability was determined using a transwell permeability assay. (B) BALB/c mice were subcutaneously injected with BSA, rMIF, rMIF and BafA1, or rMIF and CQ. The mice were sacrificed 30 min after intravenous injection of 5% Evans blue dye (200 µl). (C) BALB/c mice were intraperitoneally injected with BSA, murine rMIF, rMIF and BafA1, or rMIF and CQ. After 30 min, the mice were sacrificed, and the abdominal cavities were washed with 10 ml of PBS. The concentration of protein in abdominal washings was detected using the BCA method. n = 5. (D) HUVECs were treated with rMIF alone or with 3-MA, NAC, BafA1, or CQ for 30 min. Relative endothelial permeability was determined using the transwell permeability assay. *P<0.05, **P<0.01, ***P<0.001.

To confirm whether autophagic degradation is also involved in rMIF-induced vascular leakage, we used BafA1 and CQ to block autophagic degradation. BafA1 and CQ treatment attenuated rMIF-induced endothelial dysfunction *in vitro* (Fig. 6A). BafA1 and CQ also abolished rMIF-induced vascular leakage when subcutaneously injected (Fig. 6B) or intraperitoneally injected (Fig. 6C). These results demonstrate that autophagic degradation is a crucial step in MIF-induced vascular leakage. This phenomenon was also confirmed in primary cultured human umbilical vein endothelial cells (HUVECs), in which 3-MA, NAC, BafA1 and CQ were able to block rMIF-induced endothelial barrier dysfunction (Fig. 6D).

### ERK signaling mediates MIF-induced autophagy and vascular leakage

**Fig. 7.**
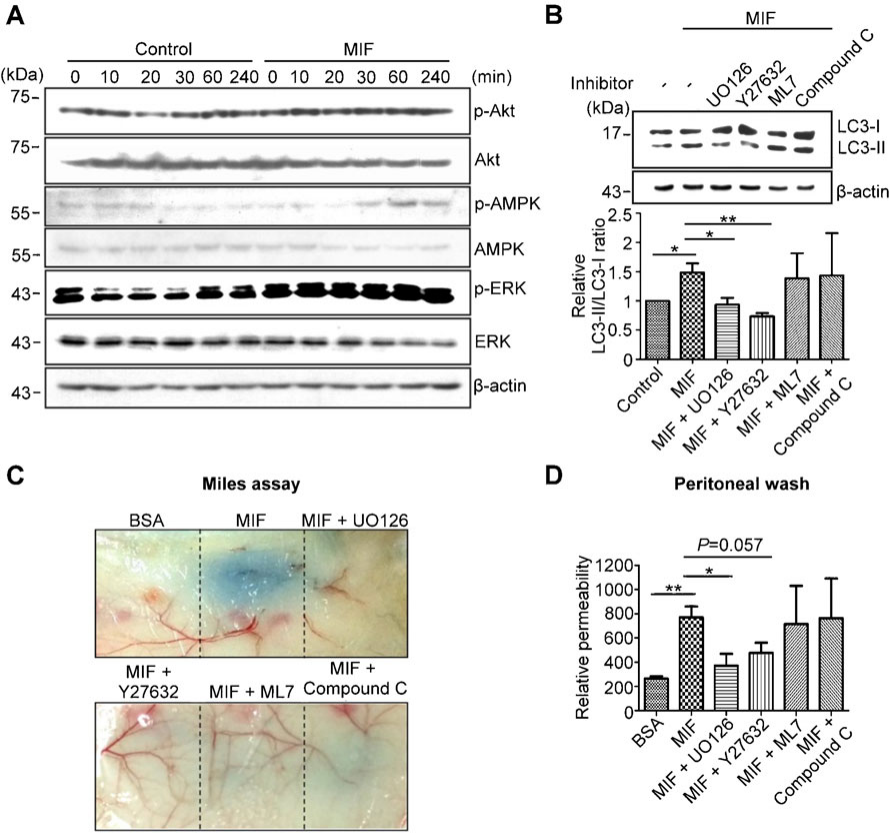
ERK mediates MIF-induced vascular leakage. (A) HMEC-1 cells were treated with or without 1 ng/ml rMIF for the indicated time periods. Relative protein levels were determined by western blotting with specific antibodies. (B) HMEC-1 cells were treated with or without 1 ng/ml rMIF, rMIF and UO126, rMIF and Y27632, rMIF and ML7, or rMIF and compound C. After 30 min, cell lysates were collected for western blotting. LC3-II to LC3-I conversion was quantified using ImageJ software. (C) BALB/c mice were subcutaneously injected with BSA, rMIF, rMIF and UO126, rMIF and Y27632, rMIF and ML7, or rMIF and compound C. The mice were sacrificed 30 min after intravenous injection of 5% Evans blue dye (200 µl). (D) BALB/c mice were intraperitoneally injected with 0.1 mg/ml BSA, 0.1 mg/ml murine rMIF, rMIF and UO126, rMIF and Y27632, rMIF and ML7, or rMIF and compound C. After 30 min, the mice were sacrificed, and the abdominal cavities were washed with 10 ml of PBS. The concentration of protein in abdominal washings was detected by BCA method. n = 4, *P<0.05, ***P<0.001.

To further elucidate the signaling pathway related to rMIF-induced autophagy that results in vascular leakage, the phosphorylation levels of kinases that are involved in autophagy induction were studied. It is known that the binding of MIF to its receptor CXCR2, CXCR4 and/or CD74 activates downstream signals including PI3K/Akt and MAPK/ERK (Lue et al., 2006; Lue et al., 2007). In addition, the MAPK/ERK pathway is an important mediator of autophagy induction in colon cancer cells (Ogier-Denis et al., 2000). During energy depletion, 5′-AMP-activated protein kinase (AMPK) activation is a main signaling pathway that initiates autophagy via inhibition of mTOR (Zoncu et al., 2011). Therefore, we compared the phosphorylation levels of Akt, AMPK and ERK after rMIF treatment. The phosphorylation level of Akt was not significantly altered after rMIF treatment, while rMIF increased the phosphorylation level of AMPK and ERK within 30 min in HMEC-1 cells (Fig. 7A), indicating that AMPK and ERK might be involved in rMIF-induced autophagy. To further determine which kinases were involved in rMIF-induced autophagy, the ERK inhibitor UO126, the Rho-associated protein kinase (ROCK) inhibitor Y27632, the myosin light chain kinase (MLCK) inhibitor ML7, or the AMPK inhibitor compound C were used to treat HMEC-1 cells together with rMIF. UO126 and Y27632 inhibited the rMIF-induced conversion of LC3 in HMEC-1 cells, indicating that ERK and ROCK activation might contribute to rMIF-induced autophagy (Fig. 7B). However, the *in vivo* study showed that when BALB/c mice were subcutaneously injected with rMIF and different inhibitors, UO126, Y27632, and ML7 significantly rescued vascular leakage, while compound C only partially reversed MIF-induced vascular leakage (Fig. 7C). On the contrary, when rMIF and these inhibitors were intraperitoneally injected, only UO126 significantly rescued protein extravasation (Fig. 7D).

## DISCUSSION

**Fig. 8.**
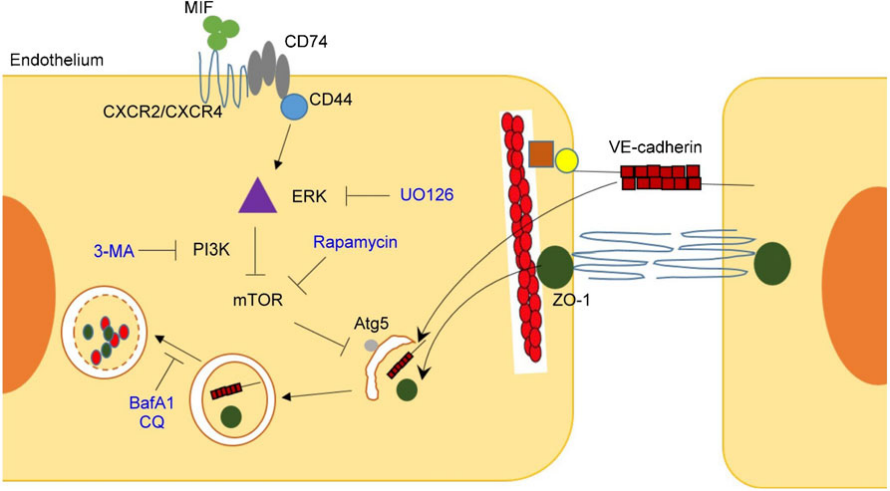
Hypothetical model of the signaling pathway through which MIF increases vascular permeability. Extracellular MIF binds to the cell surface receptor complex, which consists of CXCR2/CXCR4 and CD74. CD74 interacts with CD44, which mediates signal transduction through MAPK/ERK. ERK has been reported to repress the mTOR complex via the PI3K/Akt pathway to induce the formation of autophagosomes, which later fuse with lysosomes to become autophagolysosomes and degrade proteins. Both MIF-induced junction protein translocation and degradation could increase vascular permeability.

In the present study, we show that MIF can induce autophagy formation in endothelial cells. Autophagy mediates the degradation of the junction protein ZO-1 and VE-cadherin, thus results in an increase in vascular permeability. These effects are associated with MIF-induced ERK activation. A hypothetical model of the signaling pathway through which MIF increases vascular permeability is shown in Fig. 8.

Autophagy is widely studied in numerous fields; however, its correlation with endothelial barrier dysfunction has not yet been comprehensively investigated. One study hypothesized that the autophagy induced by alumina nanoparticles may mediate endothelial barrier disruption in the brain (Chen et al., 2013). In addition, studies of endogenous molecules also indicate that autophagy and vascular leakage are linked. For example, HMGB1 can regulate autophagy (Tang et al., 2010) and increase vascular permeability. Thrombin has also been shown to activate autophagy in the brain and increase endothelial permeability (Hu et al., 2011; Rabiet et al., 1996). Cytokines secreted during sepsis can also increase vascular permeability and induce autophagy (Chuang et al., 2012; Harris, 2011; Hofmann et al., 2002; Wolfson et al., 2011). Our data further demonstrated that an autophagy inducer (rapamycin) is sufficient to induce vascular leakage (Fig. 4). These examples strongly suggest that autophagy-induced vascular leakage is not unique to MIF.

Vascular permeability is also regulated by the expression and localization of junction proteins. For example, VEGF was shown to induce endothelial hyperpermeability through β-arrestin-dependent endocytosis of VE-cadherin (Gavard and Gutkind, 2006). TNF-α altered retinal vascular permeability in diabetes through down-regulation of ZO-1 and claudin-5 protein and mRNA, which is mediated by NF-κB activation (Aveleira et al., 2010). TNF-α has also been suggested to increase vascular permeability by reducing VE-cadherin mRNA expression (Hofmann et al., 2002). IL-1β disrupts VE-cadherin arrangement through the MYD88-ARNO-ARF6 pathway and enhances vascular permeability (Zhu et al., 2012). VEGF, TNF-α and thrombin have been shown to disrupt VE-cadherin arrangement by promoting small GTPase-mediated actomyosin contractility (Bryan et al., 2010; Huveneers et al., 2012; van Nieuw Amerongen et al., 2000; Zeng et al., 2002). The barrier function of endothelium is affected by the stability of cell-cell junctions, which is characterized by low-actomyosin tension and well-organized adhesion molecules. The contraction of actomyosin results in the misalignment of junction proteins and weakens cell-cell adhesion. The resultant gaps between the endothelial cells provide passages for fluids and small molecules and lead to vascular leakage. Our data also showed that MIF induced actin polymerization and VE-cadherin disorganization (Fig. 2A) within 30 min, indicating that MIF promoted actin rearrangement and resulted in the disorganization of junction proteins. However, in the later stages (after 60 min of MIF treatment), these junction proteins were degraded. The degradation of junction proteins occurs together with p62 and LC3 degradation 60 min after MIF treatment (Fig. 2B, Fig. 3C). Therefore, the disorganization of junction proteins is involved in the early stage of MIF-induced hyper-permeability, while the degradation of junction proteins is involved in the late stage. This result is consistent with our previous study (Chuang et al., 2011).

The binding of MIF to CXCR2, CXCR4 and/or CD74 induces the activation of PI3K/Akt and MAPK/ERK (Bernhagen et al., 2007; Schwartz et al., 2009; Lue et al., 2006; Lue et al., 2007). Both pathways have been implicated in the induction of autophagy. However, as our results showed, MIF activated ERK, but not Akt, in HMEC-1 cells (Fig. 7A). The AMPK pathway was also implicated in autophagy induction, but the activation of AMPK by MIF is cell type-specific. It has been reported that MIF negatively regulates AMPK in human non-small cell lung carcinoma cell lines (Brock et al., 2012). Other studies indicated that MIF increases AMPK activity in cardiomyocytes, hepatocytes, and endothelial cells, as shown by our data (Fig. 7A) (Heinrichs et al., 2011; Wang et al., 2013). However, inhibition of AMPK by compound C failed to effectively prevent MIF-induced autophagy and vascular leakage (Fig. 7B,D). Myosin II activation has also been proposed to regulate starvation-induced autophagy (Tang et al., 2011). In this study, we used inhibitors of these signaling pathways to explore potential pathways that are involved in MIF-induced autophagy and vascular leakage. The results from *in vitro* and *in vivo* studies showed that the ability of these inhibitors to prevent MIF-induced autophagy and vascular leakage was variable. This discrepancy may have arisen because the signaling pathways that regulate endothelial autophagy and vascular permeability in different tissues may be different. Nevertheless, the ERK inhibitor UO126 significantly reduced MIF-induced autophagy and vascular leakage both *in vitro* and *in vivo*. Therefore, it is possible that ERK activation is the major pathway involved in MIF-induced autophagy and vascular leakage of endothelial cells.

In summary, in this study, we demonstrated that inhibition of autophagic flux not only rescued MIF-induced vascular leakage in HMEC-1 cells but also in primary cultured HUVECs. Subcutaneous and intraperitoneal injection of rMIF into mice further confirmed that autophagy inhibitors could prevent MIF-induced vascular leakage *in vivo*. In septic patients, vascular leakage is a rapid and reversible process that involves apoptosis of endothelial cells, dysfunction of endothelial junctions, disruption of the glycocalyx and degradation of the extracellular matrix (Peters et al., 2003; Schmidt et al., 2012; Tressel et al., 2011). Currently, there is no specific drug that can be used to treat vascular leakage-induced edema and hypotension in sepsis. Xigris was the only drug that had been approved by the Food and Drug Administration for treating sepsis; however, it was recalled in 2011 because it increased the risk of bleeding and could not increase the survival rate. Here, we provide a model illustrating that autophagy mediates the disorganization and degradation of junction proteins in MIF-induced vascular leakage, which is in agreement with the “rapid and reversible” features of vascular leakage in acute inflammatory shock patients. After further investigation, inhibition of autophagy may serve as a potential therapy for vascular leakage in shock patients.

## MATERIALS AND METHODS

### Cells

HMEC-1 cells were cultured in MCDB 131 medium (Sigma-Aldrich, St. Louis, MO) supplemented with 10% fetal bovine serum (FBS; HyClone Laboratory, Logan, UT), 25 µg/ml epidermal growth factor (Millipore, Germany) and 25 U heparin (Sigma-Aldrich) at 37°C in a 5% CO_2_ atmosphere.

Stable clones of luciferase (Luc)-knockdown HMEC-1 cells were generated using a lentivirus-based short hairpin RNA (shRNA) system (National RNAi Core Facility, Academia Sinica, Taipei, Taiwan) targeting the sequence 5′-GCCACAACATCGAGGACGGCA-3′. The stable Atg5-silenced HMEC-1 cell line was a kind gift from Dr. Chiou-Feng Lin. Both shLuc and shAtg5 HMEC-1 cells were selected with 2 µg/ml puromycin (MDBio, Inc, Taiwan).

Primary cultured HUVECs were obtained from the Bioresource Collection and Research Center of Food Industry Research and Development Institute, Taiwan. The cells were cultured with endothelial cell growth media kits (Lonza, Switzerland).

### Recombinant MIF production

Human and mouse rMIF were produced as previously described (Chuang et al., 2011). Both were cloned and expressed in *Escherichia coli* Rosetta using the T7 polymerase-based pET-43.1a(+) vector (Novagen, Madison, WI) with the (His)_6_-tag fusion protein. After homogenization, rMIF was purified using Ni Sepharose (GE Healthcare, Sweden). The purified protein was further analyzed by SDS-PAGE and western blotting.

### Inhibitors and treatment

In *in vitro* experiments, 1 ng/ml human rMIF was applied. In *in vivo* experiments, 50 µg of murine rMIF was injected intraperitoneally or subcutaneously. As a negative control, rMIF was heat-denatured at 90°C for 10 min. To inhibit MIF activity, the MIF tautomerase inhibitor ISO-1 (50 µM; Calbiochem, La Jolla, CA) or anti-MIF polyclonal antibodies (10 µg/ml) were mixed with rMIF before treatment. The rabbit anti-MIF polyclonal antibody was purified from rMIF-immunized rabbit serum. To inhibit autophagy, 5 mM 3-MA (Sigma-Aldrich) or 5 mM NAC (Sigma-Aldrich) was used. Rapamycin (Bio Basic Inc., Amherst, NY) was added at 100 nM to induce autophagy. To block autophagy flux, 25 nM Baf A1 (Alfa Aesar, Ward Hill, MA) or 100 µM CQ (Sigma-Aldrich) was applied. The MAPK/ERK inhibitor UO126 (10 µM, Sigma-Aldrich), the ROCK inhibitor Y27632 (50 µM, Santa Cruz Biotechnology, Inc., Dallas, TX), the MLCK inhibitor ML7 (50 µM, Santa Cruz Biotechnology) and the AMPK inhibitor compound C (10 µM, Sigma-Aldrich) were used in both *in vitro* and *in vivo* experiments.

### Transwell permeability assay

For the *in vitro* permeability assay, cells (2×10^5^) were grown on a transwell insert (0.4 µm; Corning B.V. Life Sciences, The Netherlands) until a monolayer was formed. The upper chambers were reconstituted with 10% FBS-containing medium with MIF and inhibitors. At the indicated time points, the media in the upper chambers were replaced with 300 µl of serum-free media containing 4.5 µl of streptavidin-horseradish peroxidase (HRP) (R&D Systems, Inc., Minneapolis, MN). The media (20 µl) in the lower chambers were collected 5 min after adding streptavidin-HRP and assayed for HRP activity by adding 100 µl of 3,3′,5,5′-tetramethylbenzidine (TMB) substrate (R&D Systems). Color development was detected using a VersaMax microplate reader (Molecular Devices, Sunnyvale, CA) at 450 nm.

### Western blotting

For western blotting, ZO-1, VE-cadherin (BD Transduction Laboratories, Franklin Lakes, NJ), p-FRAP (p-mTOR), p-Akt1/2/3, Akt1/2/3, p-ERK, ERK, p-AMPK, AMPK, p62 (Santa Cruz), LC3 (MBL, Woburn, MA), and mTOR (GeneTex, Inc., Irvine, CA) were detected using antibodies diluted 1:1,000, followed by an HRP-conjugated anti-mouse or anti-rabbit immunoglobulin antibody diluted 1:6,000 (Leadgene Biomedical, Taiwan). β-actin antibodies (Sigma-Aldrich) were used at a 1:10,000 dilution as an internal control. Bound HRP-conjugated antibodies were detected using Luminata^TM^ Crescendo Western HRP substrate (Millipore). The results of western blotting were quantified using ImageJ software.

### Immunocytochemistry

Cell monolayers were seeded onto microscope cover glass. After 30 min of treatment, the cells were fixed in 4% paraformaldehyde for 5 min followed by three washes with PBS. The cells were then blocked with Bløk-FL noise canceling reagent (Millipore) for 1 h at room temperature. To detect VE-cadherin localization, a mouse anti-VE-cadherin monoclonal antibody (Beckman Coulter, Brea, CA) (1:200 dilution in PBS) was incubated with the cells overnight at 4°C. After three washes with TBST, the cells were treated with an Alexa 488-conjugated goat anti-mouse IgG monoclonal antibody (Invitrogen, Carlsbad, CA) (1:500 dilution) and Alexa Fluor 647 Phalloidin dye (Invitrogen) (1:1,000 dilution) for 1 h. Subsequently, three washes were performed with Tris-buffered saline-tween 20. Images were obtained using a confocal microscope (Olympus FluoView FV1000, Melville, NY).

### Transfection

To analyze ptfLC3 punctae formation, HMEC-1 cells (5×10^5^ cells) were seeded onto 6-cm cell culture dishes. The cells were transfected with the ptfLC3 construct (a gift from Dr. Tamotsu Yoshimori) using Hyfect (Leadgene Biomedical, Inc.) following the manufacturer's instructions. After incubation for 24 h, the cells were sub-cultured on glass coverslips. After monolayer formed, rMIF was then added to the cells and allowed to incubate for 1 h. Subsequently, the cells were fixed, and immunofluorescence staining was applied.

### Miles assay

Subcutaneous vascular leakage in mice was tested using a modified Miles assay (Miles and Miles, 1952). BALB/c mice (8–12 months old) were purchased and maintained at the Laboratory Animal Center of National Cheng Kung University (NCKU). The experiments were approved by the Institutional Animal Care and Use Committee of NCKU. The mice were injected intradermally with 50 µg of murine rMIF with or without inhibitors, followed by intravenous injection of 200 µl of 0.5% Evans Blue (Alfa Aesar) 5 min later. The mice were sacrificed 30 min after Evans Blue injection. The dorsal skins of the mice were photographed for observation.

### Vesicular leakage in the peritoneal cavity

The method for testing vesicular leakage in the peritoneal cavity was described previously (Chuang et al., 2011). BALB/c mice (8–12 months old) were injected intraperitoneally with 50 µg of murine rMIF, which was dissolved in 500 µl of PBS with or without inhibitors. The mice were sacrificed 30 min after the treatments. The abdominal cavity was then washed with 10 ml of PBS. The concentration of protein in abdominal washings was detected using the BCA method (Pierce Biotechnology, Rockford, IL).

### Statistical analysis

The data are expressed as the mean±standard error of the mean (SEM) of more than three independent experiments. Student's *t*-test was used to analyze the significance of differences between the experimental and control groups. *P* values <0.05 were considered statistically significant.
